# Gender Role Discrepancy Stress and COVID-19 Prevention Behaviors Among Men in the United States

**DOI:** 10.1177/08901171231152140

**Published:** 2023-01-17

**Authors:** Katelyn M. Sileo, Rebecca Luttinen, Suyapa Muñoz, Terrence D. Hill

**Affiliations:** 1 The Department of Public Health, College of Health, Community, and Policy, 12346 The University of Texas at San Antonio, San Antonio, TX, USA; 2 The Department of Demography, College of Health, Community, and Policy, 12346 The University of Texas at San Antonio, San Antonio, TX, USA; 3 The Department of Sociology, College of Health, Community, and Policy, 12346 The University of Texas at San Antonio, San Antonio, TX, USA

**Keywords:** COVID-19, health behavior, men, masculinity

## Abstract

**Purpose:**

To examine the associations between gender role discrepancy (non-conformity to socially prescribed masculine gender role norms) and discrepancy stress (distress arising from this discrepancy) on COVID-19 prevention behaviors among men, and the potential moderating effects of race/ethnicity, sexual orientation, and income on these relationships.

**Design:**

A national online survey was conducted between May and June 2021.

**Setting:**

The United States.

**Subjects:**

749 adult men residing in the United States.

**Measures:**

A scale measured gender role discrepancy and discrepancy stress. COVID-19 prevention outcomes were constructed and included self-reported vaccination status/intentions, social distancing, mask-wearing, and hand-sanitizing.

**Analysis:**

Multivariate generalized linear models were performed in SPSS.

**Results:**

Gender role discrepancy associated with greater odds of vaccination (AOR = 1.35, 95% CI = 1.02-1.78, *P* = .04), while discrepancy stress associated with lower odds of vaccination (AOR = .48, 95% CI = .35-.68, *P* < 0. 001) and mask-wearing (AOR = .54, 95% CI = .37-.79, *P* = .001) for men overall. Discrepancy stress’s negative effect on specific COVID-19 prevention behaviors was only apparent or was amplified for men in lower income brackets (vaccination, social distancing, mask-wearing), racial/ethnic minority men (vaccination), and sexual minority men (social distancing).

**Conclusion:**

This study demonstrates that gender role discrepancy stress negatively affects men’s engagement in COVID-19 prevention, particularly for men in marginalized populations.

## Introduction

Engagement in the Centers for Disease Control and Prevention’s (CDC) recommended COVID-19 prevention behaviors remain critical to controlling the pandemic globally and in the United States (U.S.), including vaccination, mask-wearing, social distancing, and other hygiene behaviors. However, large variation in vaccination and prevention behaviors by region and sub-population warrants investigation.^1,2^ One important contributing factor to variation in COVID-19 prevention engagement is gender; differences in health behavior engagement between men and women is theorized to be a driving factor of greater COVID-19 related mortality in the U.S. among males compared females, observed across pandemic wave and geographic region.^3^ Emerging research supports this hypothesis for a number of COVID-19 prevention behaviors, which are reportedly lower among men compared to women in a variety of settings and sub-populations.^4-7^ Understanding how gender influences men’s care engagement is critically important to the development of public health programming tailored to men’s needs, and in turn, to improving public health efforts for pandemic control. Thus, this study sought to examine how masculine norms affect men’s engagement in COVID-19 preventative behaviors in the United States, with a focus on gender role discrepancy stress.

Masculine norms, or the culturally grounded expectations for men’s roles, behaviors, and relationships, are central to men’s engagement in health behaviors across health outcomes.^8,9^ The socialization of men to be strong, resilient, independent, stoic, and to avoid the appearance of weakness and femininity may reduce their willingness to engage in preventative health behaviors.^8,9^ Studies in the U.S. demonstrate adherence to traditional masculine norms is generally associated with less healthcare utilization, such as lower health literacy, less HIV or sexually transmitted infection (STI) testing, and less psychological help seeking behavior.^10-12^ Several studies report conforming to traditional masculine norms associates with lower adherence to CDC-recommended COVID-19 prevention behaviors,^13-17^ but the research in this area is still limited.

Gender role discrepancy stress is a specific construct from the gender theory literature previously linked to men’s health behavior,^18^ but not yet been examined in relation to COVID-19. Reidy et al. conceptualizes and measures this phenomenon with two related constructs: gender role discrepancy (non-conformity to socially prescribed masculine gender role norms) and discrepancy stress (distress arising from perceived failure to conform to these norms).^19^ Gender role discrepancy alone is not necessarily a risk for unhealthy behaviors.^20,21^ However, strain resulting from this discrepancy can cause feelings of inadequacy, anger, and low self-esteem in men.^22,23^ It and similar measures of gender role strain positively associate with greater engagement in intimate partner violence, sexual risk behaviors, and alcohol abuse.^19,22,24,25^ Men with high discrepancy stress may engage in unhealthy behaviors to cope with feelings of inadequacy, or to compensate for them, by behaving in hyper-masculine ways.^26^ This theory aligns with the compensatory masculinity hypothesis that posits that in response to gender role threat men exaggerate their masculinity and engage in more antisocial behavior.^27^

Less research has examined gender role discrepancy stress and compensatory masculinity in relation to preventative health behaviors. However, men with high discrepancy stress may avoid COVID-19 preventive practices if they perceive these behaviors as feminine, a sign of perceived vulnerability, or counter to masculine norms of strength and self-reliance. A large body of evidence demonstrates men who adhere to traditional masculinities engage in less self-care^17^ and feel seeking and engaging in healthcare suggests weakness or femininity.^28,29^ Specific to preventative self-care, a study with African American men with diabetes reported that maintaining a public persona of strength resulted in neglect of diabetes management for some men.^30^ While research specific to COVID-19 prevention is still growing, Capraro and Barcelo^4^ reported that men in the U.S. were more likely to agree that wearing a mask was a sign of weakness and to report feeling stigmatized and ashamed when wearing masks compared to women. Research reports men scoring high on masculine “toughness” have more negative attitudes towards mask-wearing^16^ and men rating themselves as “completely masculine” are less likely to support mask-wearing and bans on gatherings of more than 10 people to prevent COVID-19 than other men.^31^ As such, for men experiencing gender role discrepancy stress, public non-compliance with COVID-19 preventive behaviors that are associated with weakness could be viewed as a display of masculine strength.

Further, the effect of gender role discrepancy stress on COVID prevention outcomes may differ across groups of men. Men in marginalized groups that have more barriers to accessing traditional markers of masculinity (e.g., success, money) could be more likely to experience discrepancy stress, and may be more likely to engage in compensatory expressions of masculinity. These differences are important to explore, given stark health disparities in COVID-19 outcomes, such as greater risk of infection, hospitalization, and death among American Indian or Alaska Native, Asian, Black, and Hispanic people compared to White people.^32^ Low socioeconomic status is intrinsically linked to racial health disparities, and also increases COVID-19 vulnerability.^33,34^ Moreover, sexual minority men have a greater prevalence of underlying health conditions associated with severe outcomes from COVID-19 than heterosexual persons.^35^ A qualitative study with young black men who have sex with men (MSM) underscores the importance of examining discrepancy stress in the context of men’s intersecting identities; the study reported how pressure to conform to heterosexual gender role expectations resulted in psychological distress and efforts to prove one’s masculinity among men.^36^ The study also outlined a number of ways that the strain arising from conflict between one’s sexual identity and cultural norms of masculinity can increase men’s HIV risk (e.g., poor self-esteem, reduced access to HIV prevention messaging, limited social support).

Using a probability-based national sample of adult men in the U.S., the primary aim of this study is to examine the role of gender role discrepancy and discrepancy stress on four COVID-19 prevention behaviors (vaccination, social distancing, mask-wearing, hand-sanitizing). We hypothesize that gender role discrepancy in itself (viewing oneself as less masculine than others) will have no association or a positive association with prevention behavior, based on prior research.^20,21^ However, we hypothesize that discrepancy stress (being concerned about being less masculine than others) will be associated with less prevention behaviors. A secondary aim of this study is to examine the potential moderating role of relevant sociodemographic variables on the relationship between discrepancy stress and these outcomes, including race/ethnicity, sexual orientation, and income. We hypothesize that racial/ethnic minority, sexual minority, and lower income men may be more affected by gender discrepancy stress than White men, heterosexual men, and higher income men, respectively. Research in this area could have important implications for reducing gender disparities in COVID-19 health outcomes by tailoring COVID-19 health promotion messaging and interventions to men.

## Methods

This study uses cross-sectional data from the 2021 Crime, Health, and Politics Survey (CHAPS) collected during the COVID-19 pandemic. CHAPS measures the social causes and consequences of various health and well-being indicators among a national probability sample of 1771 community-dwelling adults aged 18 and over living the U.S., including 806 men (biological males at birth) included in this analysis. The survey includes measures of psychosocial characteristics, religious beliefs and experiences, political views and behaviors, neighborhood conditions, experiences with crime and police, stressful life events, health behavior and health lifestyles, and sociodemographic characteristics.

Respondents were sampled from the National Opinion Research Center’s (NORC) AmeriSpeak© panel, which is representative of households from all 50 states and the District of Columbia.^37^ Between May 10, 2021 and June 1, 2021 participants were sampled and invited to complete an online survey in English; at this time in the U.S., the COVID-19 vaccine was available. The data collection process yielded a survey completion rate of 30.7% and a weighted cumulative response rate of 4.4%. The multistage probability sample resulted in a margin of error of ±3.23% and an average design effect of 1.92. The self-administered web-based survey lasted approximately 25 minutes. All respondents were offered the cash equivalent of $8.00 for completing the survey. The survey was reviewed and approved by the institutional review boards at NORC (21-05-279) and the University of Texas at San Antonio (FY20-21-29). Written informed consent was obtained from all participants.

### Measures

#### COVID-19 Prevention Behaviors

To measure the outcome of *vaccination status/intentions*, participants answered the question: “Have you been vaccinated for the coronavirus (COVID-19)?” For analysis, “Yes” and “No, but I am planning to be vaccinated” were coded as 1 (vaccinated/intending to be vaccinated) and all other responses were coded as 0 (not vaccinated/not intending to be vaccinated). In addition, participants answered questions on how often they engaged in specific COVID-19 prevention behaviors during the COVID-19 pandemic, including “How often have you attended indoor gatherings >10 people?” and “How often have you worn a face covering in public places?” Response options included: “Always,” “Very Often,” “Sometimes,” “Rarely,” and “Never.” For analysis, “Rarely” and “Never” gathering in groups were classified as 1 engaging in *social distancing*, and all other responses were classified as 0 (not social distancing). *Mask-wearing* was operationalized as 1 (“Always” and “Very Often” wearing a mask) vs 0 (all other responses).

#### Gender Role Discrepancy and Discrepancy Stress

A modified version of a scale developed by Reidy et al. (2014) measured gender role discrepancy and discrepancy stress. Three items were used to measure perceived *gender role discrepancy*, or how masculine men perceive themselves to be and others perceive them to be relative to others, such as “I am less masculine than the average guy.” (Cronbach’s alpha = .87). A 6 item sub-scale measured *discrepancy stress*, or the experience of stress men feel about being less masculine than the traditional male, such as “I wish I was interested in things that other guys find interesting” and “I worry that people judge me because I am not like the typical man” (Cronbach’s alpha = .87). Response options ranged from 1 ‘‘Strongly Disagree to 7 ‘‘Strongly Agree.” For analysis, mean scores are used, with higher scores indicating greater gender role discrepancy and discrepancy stress.

#### Demographics

The following demographic items were collected from participants and used in analysis. *Age* was a continuous variable. *Race/ethnicity* included the categories: “White, non-Hispanic,” “Black, non-Hispanic,” “Hispanic,” “Other, non-Hispanic,” “2+, non-Hispanic,” and “Asian, non-Hispanic.” *Marital Status* included the 5 categories: “Married,” “Widowed,” “Divorced,” “Separated,” “Never married,” and “Living with partner.” “*Sexual orientation* included the categories: “Straight,” “Gay,” “Bisexual,” and “Other/Don’t know.” Due to low prevalence in some cells, race/ethnicity and sexual orientation, used as covariates, were dichotomized for analysis into “White” vs “Racial/ethnic minority” and “Heterosexual” vs “Non-Heterosexual.” *Income* measured annual income, and was recategorized into 4 groups for analysis based on distribution: “less than $30,000,” “$30,000-$59,000,” “$60,000-$99,999,” and “$100,000 or more.” *Education* measured the highest level of education attained, recategorized into 4 groups for analysis based on distribution: “Less than high school, or high school graduate or equivalent,” “Vocational, tech school, some college, associates,” “Bachelor’s degree,” “Post grad study/professional degree.” *Political conservatism* was measured by a single item on political ideology, with response options ranging from 1 “Very liberal” to 5 “Very conservative” (continuous variable). Finally, the *region* of the U.S. in which the participants reside was assigned by NORC according to U.S. Census classifications, reported states, and zip codes, represented by the following categories: “Northeast,” “Midwest,” “South,” and “West.”

### Data Analysis Approach

The total sample size of participants identifying as male was 844. Due to listwise deletion of missing data, our analytic sample for each outcome was reduced as follows: vaccination (n = 746, 88% of the total eligible sample), mask-wearing (n = 746, 88% of the total eligible sample), and hand-sanitizing (n = 746, 88% of the total eligible sample) and social distancing (n = 749, 89% of the total eligible sample).

Post-stratification weights were used in analysis to reduce sampling error and non-response bias. NORC developed post-stratification weights for CHAPS via iterative proportional fitting or raking to general population parameters derived from the Current Population Survey (https://www.census.gov/programs-surveys/cps/data.html). These parameters included age, sex, race/ethnicity, education, and several interactions (age*sex, age*race, and sex*race).

All analyses were conducted in SPSS version 28. Descriptive statistics and frequencies were used to describe the sample. To identify covariates relevant to our outcomes to include in our final models, the following variables were selected based on a review of the literature and then tested in bivariate analyses against each outcome: age, race/ethnicity, sexual orientation, marital status, education, income, political conservatism, and region of the U.S. Multivariable logistic regression models, controlling for covariates if associated with any one of the outcomes at *P* < .05, were used to test associations between gender role discrepancy and discrepancy stress in separate models for the 4 COVID-19 prevention outcomes: vaccination, social distancing, mask-wearing, and hand-sanitizing. Separate models adjusted for the same covariates were then run to test interactions between the following sociodemographic variables and discrepancy stress: race/ethnicity, sexual orientation, and income. Only statistically significant (*P* < .05) interactions are presented. Odds Ratios (ORs) and Adjusted Odds Ratios (AORs) are presented for bivariate and multivariate models, respectively, and 95% confidence intervals (CIs) are presented for all models.

## Results

The average age of men in the sample was 48 years (standard deviation [SD] = 17.8). The majority of men were White non-Hispanic (62.5%) and heterosexual (91.9%), with over half of the sample married (55.9%). Just over seventy percent (70.9%) of the sample reported being vaccinated or intending to be vaccinated against COVID-19, and a similar proportion of men (68.9%) reported rarely or never gathering in groups of 10 people or more since the start of the pandemic (i.e., social distancing). When asked how often respondents wore a mask in public since the start of the pandemic, 83.4% reported wearing one often or always. Approximately two-thirds of the sample (64.9%) reported often or always hand-sanitizing since the start of the pandemic. Men’s endorsement of gender role discrepancy (mean [M] = 2.34, SD = .9) and discrepancy stress (M = 2.16, SD = .8) were similar and followed a normal distribution, indicating moderate endorsement of each construct in the overall sample. See Table 1 for more details on the sample.

**Table 1.** Sample characteristics and descriptive statistics, U.S. adult men, May-June 2021 (N = 749).

	n (%)/M (SD)
COVID-19 prevention behaviors	
Vaccinated/intending to be vaccinated	529 (70.9%)
Social distancing	516 (68.9%)
Mask-wearing	622 (83.4%)
Hand-sanitizing	484 (64.9%)
Gender role discrepancy and discrepancy stress	
Gender role discrepancy (M, SD), scale: 1-5	2.34 (.9)
Masculine discrepancy stress (M, SD), scale: 1-5	2.16 (.8)
Sociodemographic characteristics	
Age (M, SD)	48.4 (17.8)
Racial/ethnic group	
White, non-Hispanic	468 (62.5%)
Black, non-Hispanic	70 (9.3%)
Other, non-Hispanic	5 (.7%)
Hispanic	121 (16.2%)
Asian, non-Hispanic	57 (7.6%)
2+, non-Hispanic	28 (3.7%)
Sexual orientation	
Gay	25 (3.3%)
Straight	688 (91.9%)
Bisexual	14 (1.9%)
Other	22 (2.9%)
Marital status	
Married	419 (55.9%)
Widowed	35 (4.7%)
Divorced	45 (6.0%)
Separated	19 (2.5%)
Never married	190 (25.4%)
Living with partner	41 (5.5%)
Education	
Less than high-school, High-school graduate/GED recipient	272 (36.3%)
Some college/associate’s degree/vocational school	192 (25.6%)
Bachelor’s degree	164 (21.9%)
Post grad study/professional degree	121 (16.2%)
Income	
Less than $30,000	168 (22.4%)
$30,000 to under $60,000	183 (24.4%)
$60,000 to under $100,000	197 (26.3%)
$100,000 or more	201 (26.8%)
Political conservatism (M, SD), scale: 1-5	3.14 (1.09)
Region	
Northeast	121 (16.2%)
Midwest	131 (17.5%)
South	301 (40.2%)
West	196 (26.2%)

Detailed statistics for the unadjusted, bivariate models used to identify covariates of the 4 COVID-19 prevention outcomes are displayed in Table 2. Since each sociodemographic variable tested was associated at *P* < .05 level with at least one of 4 COVID-19 prevention outcomes, all variables were retained as covariates in the final multivariable models.

**Table 2.** Bivariate logistic regression testing the associations between gender role discrepancy and discrepancy stress, and sociodemographic variables, on COVID-19 prevention behaviors among U.S. adult men, May-June 2021.

	Vaccination (n = 746)			Social distancing (n = 749)			Mask-wearing (n = 746)			Hand-sanitizing (n = 746)		
	OR (95% CI)	Wald χ^2^	*p*	OR (95% CI)	Wald χ^2^	*p*	OR (95% CI)	Wald χ^2^	*p*	OR (95% CI)	Wald χ^2^	*p*
Gender role discrepancy	0.99 (0.84-1.17)	0.009	.93	1.17 (0.99-1.38)	3.57	0.06	0.84 (0.69-1.03)	2.91	0.09	0.74 (0.63-0.86)	14.36	**<0.001**
Discrepancy stress	0.67 (0.54-0.82)	14.67	<**0.001**	0.95 (0.80-1.16)	0.23	0.63	0.60 (0.47-0.77)	16.86	**<0.001**	0.66 (0.54-0.80)	17.16	**<**.**0001**
Age	1.02 (1.01-1.03)	12.81	**<0.001**	1.01 (1.00-1.02)	8.37	**0.004**	1.02 (1.01-1.03)	9.98	**0.002**	1.01 (1.00-1.02	3.12	0.08
Race/ethnicity												
Racial/ethnic minority	1.13 (0.82-1.57)	0.57	0.45	1.10 (0.80-1.51)	0.33	0.56	1.27 (0.85-1.89)	1.37	0.24	1.45 (1.06-1.98)	5.31	**0.02**
White (reference)												
Sexual orientation												
Gay, Bisexual or Other	3.62 (1.62-8.06)	9.88	**0.002**	2.65 (1.32-5.30)	7.59	**0.006**	2.56 (1.00-6.51)	3.89	0.05	1.33 (0.76-2.32)	0.98	0.32
Straight (reference)												
Marital status												
Living with partner	0.47 (0.25-0.89)	5.44	**0.02**	1.55 (0.74-3.24)	1.35	0.25	1.66 (0.63-4.36)	1.06	0.30	0.77 (0.40-1.48)	0.63	0.43
Never married	1.16 (0.79-1.69)	0.58	0.45	0.92 (0.64-1.31)	0.22	0.64	1.04 (0.66-1.62)	0.02	0.88	0.59 (0.42-0.84)	8.76	**0.003**
Separated	1.19 (0.42-3.37)	0.11	0.75	4.12 (0.94-18.07)	3.52	0.06	4.03 (0.53-30.68)	1.82	0.18	1.32 (0.47-3.74)	0.28	0.59
Divorced	1.00 (0.52-1.93)	0.00	1.00	1.27 (0.65-2.48)	0.48	0.49	0.83 (0.40-1.74)	0.25	0.62	0.64 (0.35-1.18)	2.07	0.15
Widowed	3.29 (1.14-9.51)	4.83	**0.03**	1.40 (0.63-3.07)	0.70	0.40	7.62 (1.03-56.52)	3.95	0.05	2.28 (0.93-5.63)	3.22	0.07
Married (reference)												
Education												
Post grad/professional degree	5.25 (2.86-9.64)	28.72	**<0.001**	2.51 (1.50-4.21)	12.26	**<0.001**	3.01 (1.52-5.95)	10.03	**<0.001**	1.73 (1.09-2.77)	5.30	0.02
Bachelor’s degree	2.55 (1.64-3.97)	17.05	**<0.001**	1.12 (0.75-1.67)	0.29	0.59	1.58 (0.96-2.60)	3.21	0.07	1.38 (0.92-2.07)	2.43	0.12
Some college/vocational/ tech	1.53 (1.04-2.25)	4.65	**0.031**	1.41 (0.95-2.09)	2.94	0.09	1.73 (1.07-2.82)	4.97	0.03	1.03 (0.71-1.50)	0.03	0.87
HS grad or less (reference)												
Income												
$100,000 or more	3.20 (1.92-5.36)	19.74	**<0.001**	0.97 (0.60-1.58)	0.01	0.91	2.31 (1.21-4.42)	6.43	0.011	1.21 (0.79-1.87)	0.77	0.38
$60,000-$100,000	1.11(0.72-1.72)	0.23	0.63	0.46 (0.29-0.72)	11.44	**0.001**	0.65 (0.39-1.09)	2.66	0.10	1.01 (0.66-1.54)	0.001	0.97
$30,000- $60,000	0.76 (0.49-1.16)	1.61	0.21	0.42 (0.27-0.67)	13.50	**<0.001**	0.80 (0.47-1.37)	0.66	0.42	0.86 (0.56-1.32)	0.49	0.49
< $30,000 (reference)												
Political conservatism	0.50 (0.42-0.59)	63.80	0.50	0.54 (0.46-0.64)	54.76	**<0.001**	0.49 (0.40-0.60)	48.32	**<0.001**	0.88 (0.76-1.00)	3.55	0**.**06
Region												
West	0.69 (0.39-1.23)	1.60	0.21	0.99 (0.60-1.62)	0.01	0.97	1.02 (.52-1.99)	0.003	0.95	1.07 (0.67-1.73)	0.09	0.77
South	0.33 (0.20-0.56)	17.16	**<0.001**	0.76 (0.48-1.20)	1.37	0.76	0.57 (0.31-1.02)	3.56	0.06	1.07 (0.69-1.67)	0.09	0.77
Midwest	0.64 (0.35-1.19)	1.99	0.16	1.07 (0.62-1.85)	0.67	0.80	0.77 (0.38-1.54)	0.55	0.46	0.45 (0.27-0.75)	9.46	**0.002**
Northeast (reference)												

Notes: OR= Odds Ratio; CI=Confidence Interval.

The multivariable models testing the associations between gender role discrepancy and discrepancy stress and the COVID-19 prevention outcomes are presented in Table 3. Gender role discrepancy was positively related to vaccination status/intentions, but not social distancing, mask-wearing, or hand-sanitizing. For each unit increase on the gender role discrepancy scale (perceiving oneself as not as masculine as other men), there was 35% greater odds of reporting being vaccinated or intending to be vaccinated (AOR = 1.35, 95% CI = 1.02-1.78, *P* = .04). The stress men feel about this discrepancy (i.e., discrepancy stress) was negatively associated with vaccination status and mask-wearing, but not social distancing or hand-sanitizing. In other words, men feeling stress about gender role discrepancy have 52% lower odds of being or intending to be vaccinated (AOR = .48, 95% CI = .35-.68, *P* < 0. 001) and 45% lower odds of reporting often or always wearing a mask in public (AOR = .54, 95% CI = .37-.79, *P* = .001).

**Table 3.** Multivariable logistic regression testing the associations between gender role discrepancy and discrepancy stress on COVID-19 prevention behaviors controlling for sociodemographic variables among U.S. adult men, May-June 2021.

	Vaccination (n = 746)			Social Distancing (n = 749)			Mask-Wearing (n = 746)			Hand-sanitizing (n = 746)		
	AOR (95% CI)	Wald χ^2^	*p*	AOR (95% CI)	Wald χ^2^	*p*	AOR (95% CI)	Wald χ^2^	*p*	AOR (95% CI)	Wald χ^2^	*p*
Gender role discrepancy	1.35 (1.02-1.78)	4.38	**.04**	1.25 (.97-1.60)	2.98	.08	1.00 (.73-1.37)	.00	.98	.80 (.64-1.00)	3.72	.05
Discrepancy stress	.48 (.35-.68)	18.14	**<.001**	.83 (.62-1.12)	1.50	.22	.54 (.37-.79)	10.21	**.001**	.75 (.57-.98)	4.59	**.03**
Covariates												
Age	1.03 (1.02-1.04)	16.92	**<.001**	1.02 (1.01-1.04)	12.64	**<.001**	1.03 (1.02-1.05)	14.20	**<.001**	1.00 (.99-1.01)	.31	.58
Race/ethnicity												
Racial/ethnic minority	1.39 (.92-2.11)	2.45	.12	1.02 (.70-1.47)	.01	.94	1.39 (.86-2.24)	1.83	.18	1.42 (1.00-2.03)	3.87	**.04**
White (reference)												
Sexual orientation												
Gay, Bisexual or Other	2.77 (1.05-7.33)	4.22	**.04**	1.22 (.53-2.80)	.22	.64	2.05 (.63-6.67)	1.43	.23	1.93 (.97-3.84)	3.49	.06
Straight (reference)												
Marital status												
Living with partner	.37 (.16-.84)	5.69	**.02**	1.21 (.52-2.79)	.20	.66	1.43 (.46-4.37)	.39	.53	.49 (.24-1.02)	3.68	.06
Never married	1.62 (.94-2.81)	2.99	.08	.98 (.60-1.59)	.01	.93	1.36 (.73-2.53)	.93	.34	.47 (.30-.76)	9.81	**.002**
Separated	.84 (.26-2.66)	.09	.76	3.10 (.66-14.51)	2.06	.15	2.19 (.27-17.59)	.54	.46	1.12 (.40-3.54)	.09	.76
Divorced	1.08 (.48-2.42)	.04	.85	1.33 (.61-2.88)	.51	.48	.75 (.31-1.83)	.40	.53	.59 (.30-1.15)	2.44	.12
Widowed	2.72 (.83-8.91)	2.74	.10	.85 (.35-2.07)	.14	.71	5.19 (.63-42.68)	2.35	.13	2.09 (.80-5.48)	2.25	.13
Married (reference)												
Education												
Post grad/professional degree	3.03 (1.46-6.26)	8.93	.**003**	2.24 (1.19-4.21)	6.23	**.01**	1.71 (.74-3.92)	1.56	.21	1.37 (.78-2.40)	1.19	.28
Bachelor’s degree	2.11 (1.21-3.67)	6.98	**.008**	1.07 (.65-1.73)	.06	.80	1.42 (.77-2.65)	1.25	.26	1.25 (.78-2.00)	.87	.35
Some college/vocational/tech	1.14 (.88-2.26)	2.05	.15	1.42 (.90-2.24)	2.32	.13	1.67 (.95-2.94)	3.18	.08	.92 (.61-1.40)	.14	.71
HS grad or less (reference)												
Income												
$100,000 or more	1.50 (.77-2.93)	1.41	.24	.63 (.34-1.18)	2.09	.15	1.35 (.59-3.10)	.50	.48	.82 (.48-1.41)	.51	.48
$60,000-$100,000	.72 (.41-1.25)	1.36	.24	.35 (.20-.61)	14.07	**<.001**	.42 (.22-.81)	6.77	**.009**	.82 (.50-1.35)	.61	.44
$30,000- $60,000	.56 (.32-.97)	4.29	**.04**	.33 (.20-5.72)	15.95	**<.001**	.77 (.40-1.48)	.61	.43	.90 (.56-1.47)	.68	.17
< $30,000 (reference)												
Political conservatism	.46 (.37-.56)	53.85	**<.001**	.51 (.43-.62)	51.12	**<.001**	.44 (.35-.56)	43.75	**<.001**	.82 (.70-.96)	6.05	**.01**
Region												
West	.56 (.29-1.08)	3.02	.08	.73 (.42-1.28)	1.20	.27	.93 (.44-1.96)	.04	.85	1.10 (.66-1.84)	.13	.72
South	.33 (.18-.59)	13.50	**<.001**	.76 (.46-1.27)	1.11	.29	.71 (.37-1.39)	1.00	.32	1.22 (.76-1.98)	.67	.41
Midwest	.72 (.36-1.44)	.88	.35	1.00 (.55-1.83)	.00	.99	.96 (.44-2.10)	.01	.92	.50 (.29-.86)	6.20	**.01**
Northeast (reference)												

Notes: AOR = Adjusted Odds Ratio; CI = Confidence Interval.

**Figure 1.**
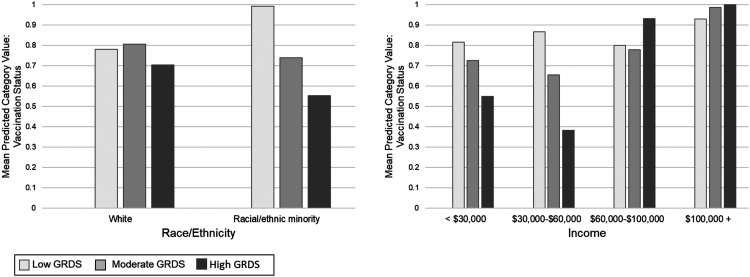
Vaccination Interactions. Graph 1: The interaction between race/ethnicity and gender role discrepancy stress on vaccination status. Graph 2: The interaction between income and gender role discrepancy stress on vaccination status. The interactions demonstrate a negative effect of gender role discrepancy stress on the odds of being/intending to be vaccinated for racial/ethnic minority men and men from lower income brackets. GRDS = gender role discrepancy stress; Low GRDS = one standard deviation below the mean or lower on the GRDS scale; Moderate GRDS = Within one standard deviation of the GRDS mean; High GRDS = one standard deviation above the mean or higher on the GRDS scale. Models control for the following covariates: age, race/ethnicity, sexual orientation, marital status, education, income, political conservatism, and geographic region.

In the models testing interactions between discrepancy stress with select covariates for each outcome, a statistically significant interaction was identified between race/ethnicity, as well as income, for vaccination status/intentions (see Figure 1). The interaction between race/ethnicity and discrepancy stress demonstrates a statistically significant negative effect of gender role discrepancy stress on the odds of being/intending to be vaccinated for racial/ethnic minority men (AOR = .27, 95% CI = .17-.44, *P* < .001) but not for White men (AOR = .70, 95% CI = .47-1.04, *P* = .08). For income, there was a statistically significant negative effect of discrepancy stress on the odds of being/intending to be vaccinated across all 4 income levels, but a greater negative effect was apparent at the lower levels of income compared to the higher levels: < $30,000/year: AOR = .46, 95% CI = .27-.79, *P* = .004; $30,000-$60,000/year: AOR = .47, 95% CI = .28-.79, *P* = .004; $60,000-$100,000/year: AOR = .55, 95% CI = .32-.93, *P* = .03; > $100,000: AOR = .44, 95% CI = .21-.90, *P* = .02.

**Figure 2.**
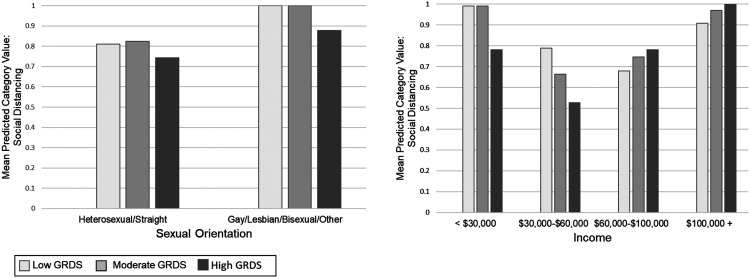
Social Distancing Interactions. Graph 1: The interaction between sexual orientation and gender role discrepancy stress on social distancing. Graph 2: The interaction between income and gender role discrepancy stress on social distancing. The interactions demonstrate a negative effect of gender role discrepancy stress on social distancing for gay/bisexual men and men from lower income brackets, but a reverse effect for men in higher income bracket (i.e., GRDS positively associated with social distancing). GRDS = gender role discrepancy stress; Low GRDS = one standard deviation below the mean or lower on the GRDS scale; Moderate GRDS = Within one standard deviation of the GRDS mean; High GRDS = one standard deviation above the mean or higher on the GRDS scale. Models control for the following covariates: age, race/ethnicity, sexual orientation, marital status, education, income, political conservatism, and geographic region.

Sexual orientation and income were identified as moderators of the relationship between discrepancy stress and social distancing (See Figure 2). For gay/bisexual men or those otherwise not identifying as heterosexual, discrepancy stress was associated with lower odds of social distancing (AOR = .40, 95% CI = .18-.88, *P* = .02), but this relationship did not hold for heterosexual men (AOR = .90, 95% CI = .66-1.22, *P* = .49). The lower levels of income were associated with lower odds of social distancing (<$30,000/year: AOR = .54, 95% CI = .33-.89, *P* = .02; $30,000-$60,000/year: AOR = .48, 95% CI = .29-.79, *P* = .004). However, for the 2 higher levels of income, the relationships trend toward the opposite direction (positive), but is statistically significant only for men earning more than $100,000 a year ($60,000-$100,000/year: AOR = 1.29, 95% CI = .79-2.10, *P* = .30; > $100,000: AOR = 2.59, 95% CI = 1.25-5.35, *P* = .01).

**Figure 3.**
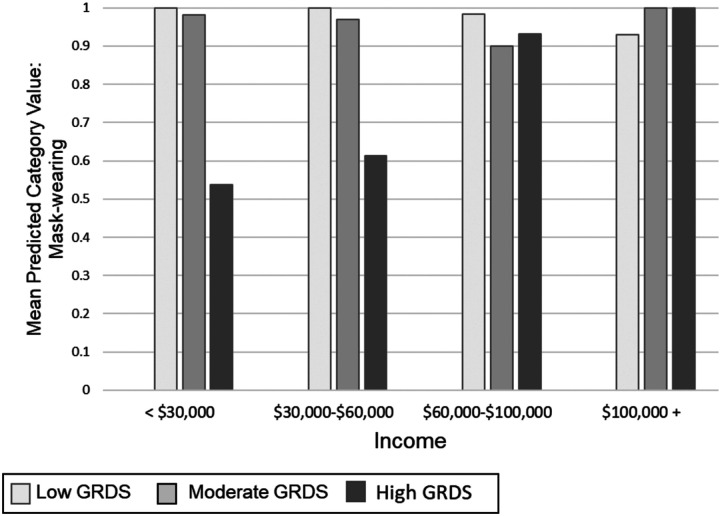
Mask-Wearing Interaction. The interaction between income gender role discrepancy stress on mask-wearing. The interaction demonstrates a negative effect of gender role discrepancy stress on mask-wearing for men from lower income brackets. The model controls for the following covariates: age, race/ethnicity, sexual orientation, marital status, education, income, political conservatism, and geographic region.

For mask-wearing, only income was identified as a statistically significant moderator (see Figure 3). No interactions were identified for hand-sanitizing. The interaction with mask-wearing demonstrated a negative effect of gender role discrepancy stress on mask-wearing for men from the lower income brackets, with a statistically significant effect at the lowest level (<$30,000/year: AOR = .34, 95 CI = .18-.67, *P* = .002) and a marginally significant effect at the second lowest level ($30,000-$60,000/year: AOR = .54, 95% CI = .29-1.00, *P* = .05). There were no statistically significant interactions between the 2 higher levels of income and discrepancy stress ($60,000-$100,000/year: AOR = .64, 95% CI = .36-1.13, *P* = .12; > $100,000: AOR = .85, 95% CI = .33-2.19, *P* = .73).

## Discussion

This study sought to understand how gender role discrepancy and discrepancy stress affect COVID-19 prevention behaviors among a nationally representative sample of U.S. adult men, and to explore potential moderators of the relationship between discrepancy stress and these outcomes. These findings add support to emerging literature that demonstrates the importance of masculinity-related constructs on men’s health behavior related to COVID-19.^13-17,38^ The findings extend the current literature by being the first to our knowledge to examine gender role discrepancy and discrepancy stress in relations to COVID-19 prevention, and by exploring whether the effect of discrepancy stress varies by race/ethnicity, sexual orientation, and income. The findings broadly suggest that gender role discrepancy was associated with better engagement in vaccination, while discrepancy stress had a negative effect on COVID-19 prevention behaviors overall. Moderation analysis revealed that discrepancy stress’s negative effect on COVID-19 prevention outcomes were only apparent or were amplified for specific subpopulations, with greater negative effects of discrepancy stress for men reporting lower income, minority men, and sexual minority men, with variation by outcome.

Viewing oneself as less masculine than others (gender role discrepancy), after the variance of discrepancy stress was controlled for, was associated with greater odds of being/intending to be vaccinated. Reidy, Brookmeyer, Gentile, et al.^34^ posit that only when one experiences stress about gender role discrepancy are they likely to adopt maladaptive behavior; others have similarly linked gender role discrepancy to positive health outcomes.^20,21,39^ Reidy and colleagues^21^ found gender discrepant boys who were not distressed about their nonconformity were less likely to engage in risky health behaviors. We reason that the men in our sample who report being less masculine than others without being stressed about it likely subscribe to less rigid gender roles overall, making these men potentially less concerned about how engaging in COVID-19 vaccination may be perceived by others.

Men that do feel stress about being less masculine than others, however, engaged in overall less COVID-19 prevention behavior, including lower odds of vaccination, mask-wearing, and hand-sanitizing in men overall. Moderation analysis revealed that the negative effect of discrepancy stress on COVID-19 prevention was present or stronger for men in lower income brackets (vaccination, social distancing, mask-wearing), racial/ethnic minority men (vaccination), and sexual minority men (social distancing). This finding contributes to research highlighting the role of intersecting identities in the differential display of masculinity and its effect on health,^8,40-42^ which is especially important given COVID-19 health disparities affecting these groups. Masculine strain may affect men of color, sexual minority men, or otherwise marginalized populations more given greater structural barriers to traditional markers of male success (e.g., status, money), such as discrimination. Bowman^43,44^ put forth a strain paradigm that can guide future research in this area, as it focuses on the nature, context, and consequences of role strain faced by individuals at risk of socially structured inequalities (i.e., racial, class, ethnic, gender). This framework guided a thematic analysis exploring male gender role strain as a barrier to African American men’s physical activity,^45^ which could be a model for future studies that dissect the context- and population-specific effects of discrepancy stress on COVID-19 prevention.

While a strength of this study is its use of a large, nationally representative sample of men in the U.S., men opting to participate in this survey could be more health-conscious than the general population and may also differ from men who do not have access to or are comfortable using the Internet/a computer. Further, these data were cross-sectional, which limits the ability to infer causation between the associations tested. Engagement in and attitudes towards COVID-19 prevention behaviors are not static, but changing overtime; this study only captures a snapshot of these relationships at a particular point in the pandemic. Local and state-specific lockdowns/mandates likely limited men’s agency in social-distancing and mask-wearing in some scenarios. Regional variation in COVID-19 transmission rates, and related risk perception, likely also affected men’s prevention engagement. These data are also limited by the self-reported nature of the variables used; social desirability could have influenced an overreporting of engagement in the COVID-19 prevention behaviors, and recall bias may have also influenced the accuracy of the self-reported outcomes. The survey was implemented early on in the pandemic, before the validation of COVID-19 behavioral scales; however, the measurement of COVID-19 prevention outcomes aligns with what has been commonly used in the literature to-date.^1,46,47^

As is a general limitation of using nationally representative samples, our study lacked the sample size to more fully examine racial/ethnic groups and the effect of sexual orientation beyond dichotomized variables. The authors acknowledge the limitations of treating diverse racial/ethnic and sexual minority men as homogenous populations, as well as the problems inherent in deferring to White/heterosexual as the automatic “reference” group. However, given the limited literature that examines these moderators at all, this approach allowed for preliminary testing of these relationships and can serve as evidence for the need for more research that fully examines these variables.

Programs to engage men in health promotion including COVID-19 prevention can benefit from understanding and incorporating the role of gender in men’s health seeking behavior into programming.^48,49^ Strategies found effective for engaging men in other health services, such as gender-sensitive staff training and the use of gender-sensitive language in public health campaigns, could be incorporated into COVID-19 prevention programming.^50^ Together with emerging research with similar aims,^13,14^ our study has implications for the tailoring of outreach messaging to engage men in COVID-19 prevention services. This might include deconstructing toxic masculine norms that reinforce the idea that vaccination, masking-wearing, or engaging in other protective behaviors as feminine or weak, while building content around how COVID-19 prevention can enhance positive masculine traits (e.g., responsibility and strength).^51^ Our findings suggest that gender transformative programming tailored specifically for low income and racial/ethnic and sexual minority men experiencing discrepancy stress may be especially important, which could be a strategy to reduce health disparities experienced by these groups. Future research can also explore our research questions in settings outside of the U.S. where cultural manifestations of gender norms may differ from the present study, but still shape men’s health seeking behaviors.^52-54^ The effect of COVID-19 prevention interventions tailored to vulnerable populations in global settings^55,56^ could be enhanced by incorporating a gender transformative approach.

## Conclusion

Engagement in COVID-19 prevention behaviors is critical to reducing the burden of COVID-19. This study demonstrates the importance of gender role discrepancy and discrepancy stress in engagement in these behaviors among U.S. men. Men who viewed themselves as not as masculine as other men were more likely to vaccinate or intend to vaccinate. However, men that felt stress about their non-conformity to masculine social standards were less likely to engage in COVID-19 prevention behaviors. This study points to the importance of tailored public health messaging and interventions to engage men in COVID-19 prevention by deconstructing harmful masculine norms and reducing discrepancy stress. Given the study’s finding that the effect of discrepancy stress is greater for men in marginalized populations, interventions may be particularly needed for low income men, and racial/ethnic and sexual minority men who are disproportionately affected by COVID-19. Future research should continue to explore the context- and population-specific relationship between gender role discrepancy and discrepancy stress on COVID-19 health behaviors and outcomes.

**SO WHAT?**

**What is already known on this topic?**

Prior research reports that men who feel stress about not meeting societal gender role expectations (i.e., gender role discrepancy stress) may engage in unhealthy behavior as compensation, and fail to engage in preventative health behaviors if doing so is viewed as counter to prevailing masculine norms.

**What does this article add?**

This study adds support for gender role discrepancy stress’s negative effect on men’s engagement in COVID-19 prevention behaviors, which may be greater for men with lower income, racial/ethnic minority men, and sexual minority men.

**What are the implications for health promotion practice or research?**

These findings can inform the development of tailored public health messaging and interventions to engage men in COVID-19 prevention by deconstructing harmful masculine norms, which may be especially needed for men in marginalized populations.
